# Use of the Delta Neutrophil Index as a Prognostic Factor of Mortality in Patients with Spontaneous Bacterial Peritonitis: Implications of a Simple and Useful Marker

**DOI:** 10.1371/journal.pone.0086884

**Published:** 2014-01-23

**Authors:** Tae Seop Lim, Beom Kyung Kim, Jong Wook Lee, Young Ki Lee, Sooyun Chang, Seung Up Kim, Do Young Kim, Sang Hoon Ahn, Kwang-Hyub Han, Chae Yoon Chon, Jun Yong Park

**Affiliations:** 1 Department of Internal Medicine, Yonsei University College of Medicine, Seoul, Republic of Korea; 2 Institute of Gastroenterology, Yonsei University College of Medicine, Seoul, Republic of Korea; 3 Liver Cirrhosis Clinical Research Center, Seoul, Republic of Korea; 4 Department of Laboratory Medicine, Incheon Baek Hospital, Incheon, Republic of Korea; University of Cincinnati, United States of America

## Abstract

**Background:**

Spontaneous bacterial peritonitis (SBP) is a common and life-threatening infection in patients with advanced cirrhosis. The prognostic value of a novel marker, the delta neutrophil index (DNI), was investigated relative to mortality in patients with SBP.

**Materials & Methods:**

Seventy-five patients with SBP were studied from April 2010 to May 2012. DNI at initial diagnosis of SBP was determined and compared with 30-day mortality rates.

**Results:**

Of the patients, 87.7% were men, and the median age of all patients was 59.0 yrs. The area under the receiver-operating characteristic (ROC) curve of DNI for 30-day mortality was 0.701 (95% confidence interval [CI], 0.553–0.849; *p* = 0.009), which was higher than that of C-reactive protein (0.640, 95% CI, 0.494–0.786; *p* = 0.076) or the model for end-stage liver disease score (0.592, 95% CI, 0.436–0.748; *p = *0.235). From the ROC curve, with the sum of sensitivity and specificity, the cutoff value of DNI was determined to be 5.7%. In the high-DNI group (DNI ≥5.7%), septic shock and 30-day mortality were more prevalent compared with the low-DNI group (84.2% vs. 48.2%, *p* = 0.007; 57.9% vs. 14.3%, *p*<0.001, respectively). Patients with an elevated DNI had a higher risk of 30-day mortality compared with those with a low DNI (4.225, 95% CI, 1.631–10.949; *p = *0.003).

**Conclusion:**

A higher DNI at the time of SBP diagnosis is an independent predictor of 30-day mortality in patients with SBP.

## Introduction

In patients with advanced cirrhosis, spontaneous bacterial peritonitis (SBP) is a common and life-threatening infection that requires prompt recognition and treatment. It is characterized by the presence of >250 polymorphonuclear cells (PMN)/mm^3^ in ascites in the absence of an intra-abdominal source of infection [1], [2]. Despite advances in the knowledge of bacterial cirrhosis pathogenesis and developments of appropriate treatment strategies, the mortality rate of SBP remains at 20% [3]–[5].

Studies have attempted to establish reliable criteria that are useful for the diagnosis and management of bacterial infection. But for patients with cirrhosis, the prognostic capabilities of conventional parameters such as systemic inflammatory response syndrome (SIRS) and C-reactive protein (CRP) are relatively limited [6]. It is difficult to assess SIRS in cirrhotic patients because of hypersplenism-induced neutropenia, increased heart rate associated with hyperkinetic circulatory syndrome, and/or hyperventilation caused by hepatic encephalopathy [7]. Furthermore, since CRP is produced predominantly by hepatocytes [8], patients with liver failure could already present with attenuated production regardless of infection.

During stress or infection, immature neutrophils enter the circulation. Termed “left-shift,” this manifestation is defined as an elevated ratio of immature granulocytes to total granulocytes [9]. It can be a useful marker of infection in clinical practice, but a more reliable and reproducible determining factor may be required. Recent technological advances have led to modern automated cell analyzers that can provide information on leukocyte differentials based on the nuclear lobularity of white blood cells (WBCs) and cytochemical myeloperoxidase (MPO) reaction [10], [11].

The delta neutrophil index (DNI), the difference between the leukocyte differentials computed in the MPO channel and those calculated in the nuclear lobularity channel [12], is significantly associated with disseminated intravascular coagulation scores, a positive blood culture rate, and mortality in patients with suspected sepsis [12]. Some studies have reported that, compared with WBC or CRP levels, DNI is a more useful marker for predicting mortality in patients with sepsis [13]–[15]. For patients with advanced cirrhosis who have been diagnosed with SBP, little is known about the clinical usefulness of DNI in evaluating infection severity of about how it relates to overall mortality. Therefore, this investigation focused on DNI values in patients with advanced cirrhosis who were treated for SBP and evaluated the clinical utility of DNI as a prognostic indicator of mortality.

## Patients and Methods

### Patients

143 consecutive patients diagnosed with SBP and admitted to Severance Hospital from April 2010 to May 2012 were retrospectively studied. The following patients were subsequently excluded from the study: 40 patients with hepatocellular carcinoma, six patients who had other cancers, and 22 patients whose ascites was caused by either pancreatitis or tuberculosis or whose culture results were suggestive of polymicrobial secondary bacterial peritonitis. After these exclusions, 75 cirrhotic patients with SBP were enrolled in the study. Liver cirrhosis was diagnosed based on histological, clinical, biochemical, or morphological results. Study protocol followed the ethical guidelines of the 1975 Declaration of Helsinki. Written informed consent was obtained from each participant or a responsible family member after the procedure and possible complications were fully explained.

### Diagnosis and Treatment of SBP

SBP diagnosis required ascitic fluid to have a polymorphonuclear (PMN) leukocyte count >250 cells/mm^3^ [1], [2]. Paracentesis was performed, and the extracted peritoneal fluid was sent for PMN count and culture study. Patients were initially treated with intravenous cefotaxime, but broad-spectrum antibiotics such as piperacillin–tazobactam or carbapenem were used in patients with septic shock based on hospital guidelines for SBP treatment [16]. Blood sampling for DNI value and culture study was performed prior to administration of antibiotics. Multi-drug resistant (MDR) bacteria were defined as organisms resistant to one or more kind of antibiotics, which included methicillin-resistant *Staphylococcus aureus* and extended-spectrum β-lactamase (ESBL)-producing *Escherichia coli* [17]. After the bacterium was isolated in the culture study, we decided to change or continue antibiotics according to its sensitivity. For example, if ESBL-producing *E.coli* was isolated in ascitic fluid, we changed the antibiotics to carbapenem. In addition, glycopeptides such as vancomycin or teicoplanin were added if methicillin-resistant gram-positive bacteria were isolated.

### Definition of Other Clinical Conditions

Community-acquired SBP was defined as diagnosis at ≤48 h of hospitalization, whereas nosocomial SBP was categorized as diagnosis >48 h from admission [18]. Septic shock was defined as sepsis-induced hypotension with a systolic arterial pressure <90 mmHg or mean arterial pressure <60–65 mmHg that persisted despite adequate fluid resuscitation. SIRS was defined as the coexistence of two or more of the following conditions resulting from infection: (1) temperature >38°C or <36°C; (2) heart rate >90 beats/min; (3) respiratory rate >20 breaths/min or PaCO_2_<32 mmHg; and (4) WBC count >12000 cells/mm^3^ or <4000 cells/mm^3^ [19]. Acute renal failure (ARF) was defined as a serum creatinine level >1.5 mg/dL in patients without pre-existing renal dysfunction or increase of more than 50% in patients with pre-existing renal dysfunction [16].

### Assessment of DNI

Blood samples were analyzed at the time of SBP diagnosis, and an automatic cell analyzer (ADVIA 2120 Hematology System, Siemens Healthcare Diagnostics, Forchheim, Germany) was used to calculate DNI [12]. This hematologic analyzer is flow cytometry-based and analyzes WBC by both a MPO channel and a lobularity/nuclear density channel.

After red blood cell lysis, the tungsten–halogen-based optical system of the MPO channel measured cell size and stain intensity in order to count and differentiate granulocytes, lymphocytes, and monocytes based on their size and MPO content. Next, the laser diode-based optical system of the lobularity/nuclear density channel counted and classified the cells according to size, lobularity, and nuclear density.

The resulting data were inserted in the following formula to determine DNI:

DNI = (neutrophil subfraction and eosinophil subfraction measured in the MPO channel) − (PMN subfraction measured in the nuclear lobularity channel).

### Statistical Analysis

The major goal of this study was to predict 30-day mortality rates based on DNI. Continuous variables were compared using the Mann–Whitney *U*-tests. Chi-squared or Fisher’s exact tests were used for categorical variables. To assess the diagnostic performance of DNI and other parameters, receiver operating characteristic (ROC) curves were constructed, and the areas under the ROC curves (AUROC) were calculated. Next, the sensitivity, specificity, positive predictive value (PPV), and negative predictive value (NPV) were calculated using the ROC curves. The optimal cutoff value of DNI to predict 30-day mortality was determined using the Youden index method, which defines the cutoff in terms of the maximal sum of sensitivity and specificity. Prognostic factors for mortality were evaluated using univariate analysis and then univariate predictors (*p*<0.05) were entered into multivariate Cox proportional hazard analyses and adjusted hazard ratios with 95% confidence intervals (CIs) were also calculated. A probability (*p*) level of 0.05 was chosen for statistical significance, and statistically significant variables were included in multivariate analysis. Furthermore, Kaplan Meier analyses evaluated 30-day mortality in the high- and low-DNI groups. Statistical analyses were performed using SPSS (version 18.0, SPSS Inc., Chicago, IL, USA).

## Results

### Population Baseline Characteristics

Baseline characteristics are presented in Table 1. A total of 75 patients of the original 143 selected were deemed eligible for this study. The median age of the patients studied was 59.0 yr, and 87.7% were male. Eleven patients (14.7%) had previous SBP history. The most common etiology of cirrhosis was chronic hepatitis B virus infection (*n* = 43, 57.3%). The median value of DNI at the time of SBP diagnosis was 3.2%, and values from 0% to 56.1%. Most patients were scored as Child-Pugh stage C (*n* = 60, 80%), and the median model for end-stage liver disease (MELD) score was 19.0. The proportion of patients with positive ascites culture, bacteremia, SIRS, and septic shock was 53.3% (*n* = 40), 36.0% (*n* = 27), 82.7% (*n* = 62), and 57.3% (*n* = 43), respectively. Thirty-day mortality occurred in 25.3% (*n* = 19) of patients.

**Table 1:** Baseline characteristics of patients with SBP.

Variables	Total (*n* = 75)
Male gender (%)	65 (87.7%)
Age, years	59.0 (38.0–82.0)
History of previous SBP (%)	11 (14.7%)
Etiology of liver cirrhosis	
HBV (%)/HCV (%)/Alcohol/Others	43 (57.3%)/10 (13.3%)/14 (18.6%)/8 (10.7%)
Community acquired SBP/Nosocomial SBP	54 (72.0%)/21 (28.0%)
ARF	20 (26.7%)
WBC count, per mm^3^	7,840 (1230–28670)
DNI, %	3.2 (0.0–56.1)
CRP, mg/L	61.9 (4.5–205.5)
Albumin, g/dL	2.4 (1.5–3.3)
Total bilirubin, mg/dL	3.6 (0.5–34.0)
Creatinine, mg/dL	1.4 (0.5–6.7)
Prothrombin time, INR	1.4 (1.1–3.1)
Na (mEq/L)	132.0 (118.0–146.0)
Child Pugh stage (B (%)/C (%))	15 (20.0%)/60 (80.0%)
Child Pugh score	11.0 (8.0–14.0)
MELD score	19.0 (8.0–35.0)
Norfloxacin prophylaxis	5 (6.6%)
Positive ascitic fluid culture (%)	40 (53.3%)
MDR bacteria in ascitic fluid culture (%)	11 (14.7%)
Bacteremia (%)	27 (36.0%)
SIRS (%)	62 (82.7%)
Septic shock (%)	43 (57.3%)
30-day mortality	19 (25.3%)

Data are reported as median (range) or number (%).

SBP, spontaneous bacterial peritonitis; HBV, Hepatitis B virus; HCV, Hepatitis C virus; ARF, acute renal failure; WBC, white blood cell count; DNI, delta neutrophil index; CRP, C-reactive protein; MELD, model for end stage liver disease; MDR, multi-drug resistant; SIRS, systemic inflammatory response syndrome.

Microbiological findings of the ascitic fluid are summarized in Table 2. The most common organism found was *E. coli* (*n* = 13, 32.5%), followed by *Klebsiella pneumoniae* (*n* = 7, 17.5%). Of 40 patients with positive ascites culture, the number of patients with MDR bacteria was 11 (27.5%).

**Table 2:** Organisms isolated in ascites.

Organisms	Total (*n* = 40), %
*E. coli*	13 (32.5%)
*Klebsiella pneumoniae*	7 (17.5%)
*Enterobacter cloacae*	4 (10.0%)
*Enterococcus facium*	4 (10.0%)
*Aeromonas hydrophila*	3 (7.5%)
*Streptococcus mitis*	2 (5.0%)
*Staphylococcus aureus*	2 (5.0%)
*Citrobacter freundii*	2 (5.0%)
*Listeria monocytogenes*	2 (5.0%)
*Sphingomonas paucimobilis*	1 (2.5%)

### Usefulness and Accuracy of DNI as a Prognostic Factor of SBP

**Figure 1:**
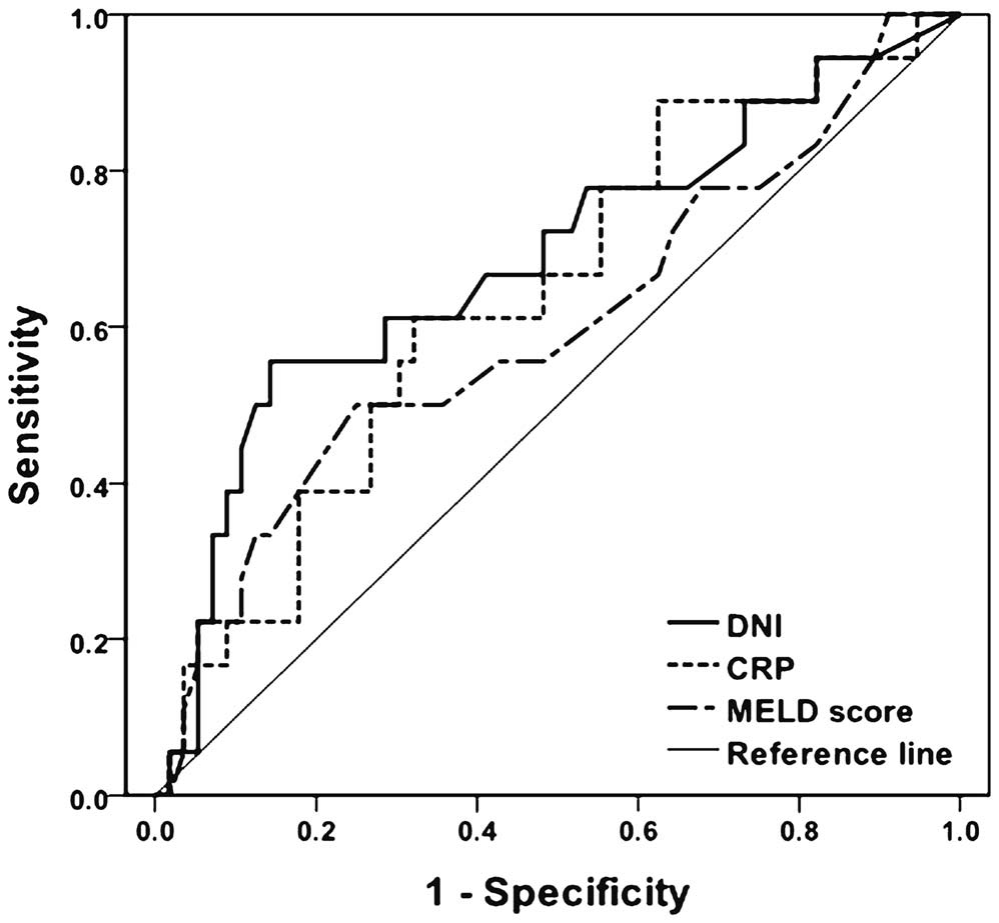
Receiver operating characteristic (ROC) curve using DNI at the onset of SBP for 30-day mortality. The area under the curve was 0.701 (95% CI, 0.553–0.849, *p* = 0.009) for DNI, 0.640 (95% CI, 0.494–0.786, *p* = 0.076) for CRP, and 0.592 (95% CI, 0.436–0.748, *p* = 0.235) for the MELD score.

To evaluate the ability of DNI to predict 30-day mortality, a ROC curve was constructed (Fig. 1). The area under the ROC curve of DNI for 30-day mortality was 0.701 (95% CI, 0.553–0.849; *p* = 0.009). This was higher than that for CRP (0.640, 95% CI, 0.494–0.786; *p* = 0.076) or the MELD score (0.592, 95% CI, 0.436–0.748; *p* = 0.235). The optimal cutoff value of DNI, obtained from the Youden index, was 5.7%, with sensitivity, specificity, PPV, and NPV values of 57.9%, 85.7%, 57.9%, and 85.7%, respectively.

### Comparisons of Variables Divided by Optimal Cutoff Value

**Figure 2:**
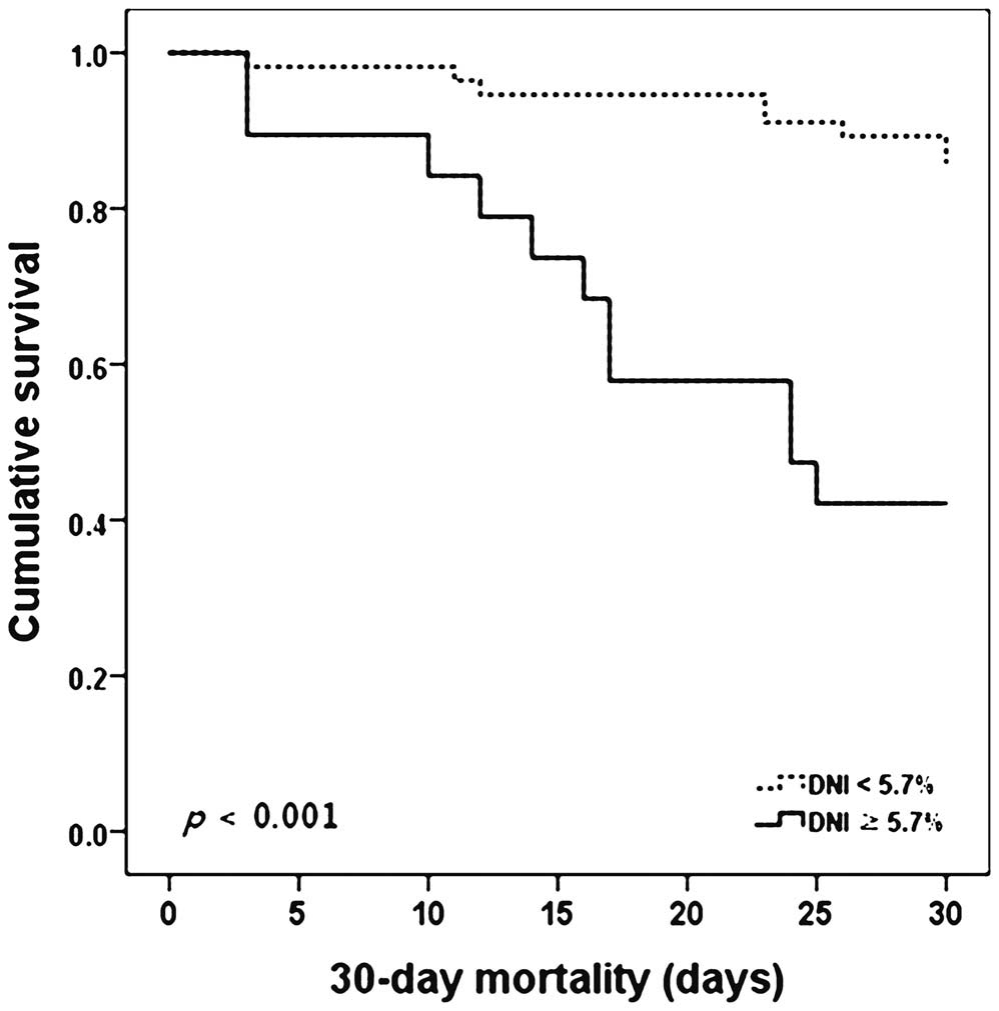
Kaplan–Meir plots for cumulative 30-day mortality in patients with SBP using the cutoff value of DNI.

Clinical and laboratory variables in the high- (≥5.7%) and low-DNI (<5.7%) groups are compared in Table 3. In the high-DNI group, septic shock and 30-day mortality occurred at greater frequency than in the low-DNI group (84.2% vs. 48.2%, *p* = 0.007). The CRP, MELD, bacteremia, and SIRS levels were all elevated in the high-DNI group, but the differences were not statistically significant. The 30-day mortality rate was significantly higher in patients with a DNI >5.7% at the onset of SBP (57.9% vs. 14.3%, *p*<0.001) (Fig. 2).

**Table 3:** Comparison of variables according to DNI cutoff value.

Variables	DNI <5.7%(*n* = 56)	DNI ≥5.7%(*n* = 19)	*p*-value	
Male gender (%)	50 (89.3%)	15 (78.9%)	0.262	
Age, years	60.0 (38.0–82.0)	58.0 (40.0–74.0)	0.884	
History of previous SBP (%)	7 (12.5%)	4 (21.1%)	0.455	
Etiology of liver cirrhosis				
HBV (%)/HCV (%)/Alcohol/Others (%)	33 (58.9%)/5 (8.9%)/10(17.9%)/8 (14.3%)	10 (52.6%)/5 (26.3%)/4(21.1%)/0 (0.0%)		
Community acquired SBP/Nosocomial SBP	37 (66.1%)/19 (33.9%)	15 (78.9%)/4 (21.1%)	0.293	
ARF	12 (21.4%)	8 (42.1%)	0.078	
WBC count, per mm^3^	7450 (1490.0–28670.0)	9390 (1230.0–20000.0)	0.893	
DNI, %	2.3 (0.0–5.5)	16.0 (5.9**–**56.1)	<0.001	
CRP, mg/L	58.6 (4.5–205.5)	64.0 (5.7–195.7)	0.345	
Albumin, g/dL	2.4 (1.5–3.3)	2.2 (1.8–3.0)	0.082	
Total bilirubin, mg/dL	3.6 (0.5–34.0)	4.2 (1.0–19.6)	0.626	
Creatinine, mg/dL	1.3 (0.5–6.7)	1.7 (0.6–4.2)	0.145	
Prothrombin time, INR	1.4 (1.1–3.0)	1.5 (1.0–3.1)	0.214	
Na (mEq/L)	132.0 (118.0–146.0)	129.0 (120.0–140.0)	0.166	
Child Pugh stage (B (%)/C (%))	12(21.4%)/44 (78.6%)	3 (15.8%)/16 (84.2%)	0.747	
Child Pugh score	11.0 (8.0–13.0)	11.0 (9.0–14.0)	0.110	
MELD score	19.0 (8.0–34.0)	22.0 (13.0–35.0)	0.059	
Norfloxacin prophylaxis	4 (7.1%)	1 (5.3%)	1.000	
Positive ascitic fluid culture (%)	29 (51.8%)	11 (57.9%)	0.645	
MDR bacteria in ascitic fluid culture (%)	8 (14.3%)	3 (15.8%)	1.000	
Bacteremia (%)	18 (32.1%)	9 (47.4%)	0.232	
SIRS (%)	44 (78.6%)	18 (94.7%)	0.164	
Septic shock (%)	27 (48.2%)	16 (84.2%)	0.007	
30-day mortality	8 (14.3%)	11 (57.9%)	<0.001	

Data are presented as median (range) or number (%).

SBP, spontaneous bacterial peritonitis; HBV, hepatitis B virus; HCV, hepatitis C virus; ARF, acute renal failure; WBC, white blood cell count; DNI, delta neutrophil index; CRP, C-reactive protein; MELD, model for end stage liver disease; MDR, multi-drug resistant; SIRS, systemic inflammatory response syndrome.

Univariate Cox proportional hazard analysis demonstrated that a DNI greater than 5.7% (univariate hazard ratio, 5.496 [2.198–13.746]; *p*<0.001) and the presence of septic shock (univariate hazard ratio, 4.544 [1.323–15.606]; *p* = 0.016) were unfavorable risk factors with respect to 30-day mortality in patients with SBP (Table 4). In the multivariate Cox proportional hazard analysis, a DNI greater than 5.7% was the only independent risk factor for 30-day mortality (adjusted hazard ratio, 4.225 [1.631–10.949]; *p* = 0.003).

**Table 4:** Cox proportional hazard analysis for 30-day mortality.

	Univariate analysis	Multivariate analysis	
	*p*-value	*p*-value	Hazard ratio (95% CI)
Male gender	0.259		
Age	0.979		
Nosocomial SBP	0.593		
ARF	0.273		
DNI ≥5.7%	<0.001	0.003	4.225 (1.631–10.949)
CRP	0.064		
Child score	0.539		
MELD score	0.148		
MDR bacteria in ascitic fluid culture	0.633		
Bacteremia	0.883		
SIRS	0.160		
Septic shock	0.016	0.086	0.086 (0.852–11.047)

CI, confidence interval; ARF, acute renal failure; DNI, delta neutrophil index; CRP, C-reactive protein; MELD, model for end stage liver disease; SIRS, systemic inflammatory response syndrome.

## Discussion

The present study demonstrates that DNI can be a useful prognostic factor for 30-day mortality in patients with SBP. There is no “gold standard” to detect sepsis early, and blood culture results are usually reported after at least 48 h. On the other hand, because levels of immature granulocytes, such as promyelocytes, metamyelocytes, and myelocytes are known to increase in infectious conditions [9], [20], it was investigated as a predictor of sepsis in several studies [21], [22]. In previous studies, the proportion of immature granulocytes correlated better with positive blood culture results and infection compared to the WBC count [21]. Furthermore, in another report, immature granulocytes was suggested as a predictor of neonatal sepsis [22]. However, it is difficult to measure immature granulocytes accurately, and their diagnostic value remains controversial. To overcome these limitations, DNI, which is the difference between the leukocyte differentials assayed in the MPO channel and those measured in the nuclear lobularity channel, was initially designed as a reliable and reproducible method to reflect immature granulocytes in circulating blood [12]. Because complete blood count is routinely evaluated in patients suspected of SBP, DNI can be easily calculated [15]. Although some studies on SBP have evaluated prognostic factors such as renal insufficiency, type of organism, bacteremia, and MELD score [23]–[31], no standard marker has been determined to predict SBP mortality. CRP [32] and SIRS [33] are common diagnostic parameters suggested for use as prognostic markers for SBP, but their values diminish when considering cirrhotic patients [6]. Although DNI has been suggested to predict mortality in other infectious conditions [12]–[15], [34], no reports have estimated the prognostic value of DNI in cirrhotic patients with SBP.

Compiled data showed that the area under a ROC curve of DNI for 30-day mortality was higher than that for CRP or MELD score. Furthermore, the optimal cutoff value of DNI was identified as 5.7%. Univariate analyses found that a DNI >5.7%, combined with the presence of septic shock, was a significant predictor of 30-day mortality in patients with SBP. Subsequent multivariate analyses revealed that a DNI >5.7% was the only risk factor necessary to predict 30-day mortality. Thus, patients with SBP who show DNI values greater than 5.0% should be managed very carefully.

Third-generation cephalosporins have been recommended as the first line of antibiotic treatment for SBP. However, extended-spectrum empirical antibiotics such as carbapenems and piperacillin/tazobactam may be considered in the high-DNI group, as recent guidelines have recommended them for use in patients with nosocomial SBP [16]. Although septic shock occurred more frequently in the high-DNI group, as is consistent with other studies [12], [13], [15], [21], [34], SIRS did not differ between the two groups. This is presumably because SIRS does not reflect well the infectious condition in cirrhotic patients due to factors such as baseline neutropenia and beta blocker use [7]. Under this hypothesis, one can raise the question whether there exists any influence of neutropenia on the DNI value and its prognostic role. In a similar study, Pyo et al. [35] investigated the role of DNI in the discrimination between disease flare-up and infection in patients with systemic lupus erythematosus patients in whom leucopenia are observed in some patients and leukocytosis are also frequently observed in other patients because of glucocorticoid usage [36], [37], indicating that DNI reflects the proportion of immature granulocytes regardless of WBC count and can better reflect infection than WBC count which can be affected by other conditions without infection. Likewise, leucopenia is common also in cirrhotic patients. Therefore, DNI may be a useful indicator especially in cirrhotic patients with leucopenia. To confirm this novel suggestion, further prospective study should be performed.

Recent reports have suggested that the MELD score could predict mortality in patients with SBP [29]–[31]. However, in this study, the MELD score was unable to predict 30-day mortality in either univariate or multivariate Cox proportional hazard analyses. This may be for several reasons. First, 80% of the patients enrolled in this study were categorized as Child-Pugh class C, so there may be no significant difference in underlying liver function among patients with advanced cirrhosis. Second, because MELD scores are commonly used as a 3-month mortality indicator in patients awaiting liver transplantation [38], it may not be possible to determine accurate associations between MELD scores and infection-related, short-term mortality.

ARF has been known to be a risk factor for acute-on-chronic liver failure in recent studies [39], but in our study, it had no effect on 30-day survival. We believe that this phenomenon is a type 2 error caused by the small sample size (i.e., only 20 patients with ARF). Although there is no statistical significance in the incidence of ARF between the two groups, the high DNI group, which was the independent predictor of 30-day mortality in our study, still showed a trend toward a higher incidence of ARF compared with the low DNI group (42.1% vs. 21.4%). Therefore, we believe that ARF may affect 30-day mortality of SBP in a larger sample size.

The connections among SIRS, multi-organ failure, and mortality have yet to be determined. Some studies have suggested that when inflammatory stress is superimposed on baseline cirrhosis, severe hemodynamic derangements may occur secondary to the accentuation of portal hypertension and reduction in hepatic blood flow [40]. This results in an increased concentration of asymmetric dimethylarginine, an endogenous nitric oxide synthase inhibitor [41]. Mediators of SIRS such as interleukin-6, interleukin-1ß, tumor necrosis factor-α, and nitric oxide may modulate hepatic encephalopathy in cirrhotic patients [42]. More recently, cirrhotic patients with SIRS were reported to exhibit marked changes in the functional capacity of albumin due to the accumulation of oxidatively modified albumin [43].

There are several limitations to this study. First, it was a retrospective study based on a small population of patients who were all treated at a single location. Second, prognosis and mortality did not take into account variations that may have existed due to the different antibiotics being administered for treatment. Moreover, because only short-term mortality was evaluated, it is unknown whether DNI can predict long-term mortality in SBP as well.

In conclusion, DNI at diagnosis of SBP is a useful prognostic factor for the determination of 30-day mortality. Patients with high DNI level should be cautiously monitored, and treatment strategies should be appropriately adapted for their future needs.
